# Microarray-Based Transcriptomic Analysis of Differences between Long-Term Gregarious and Solitarious Desert Locusts

**DOI:** 10.1371/journal.pone.0028110

**Published:** 2011-11-23

**Authors:** Liesbeth Badisco, Swidbert R. Ott, Stephen M. Rogers, Thomas Matheson, Dries Knapen, Lucia Vergauwen, Heleen Verlinden, Elisabeth Marchal, Matt R. J. Sheehy, Malcolm Burrows, Jozef Vanden Broeck

**Affiliations:** 1 Department of Animal Physiology and Neurobiology, Katholieke Universiteit Leuven, Leuven, Belgium; 2 Department of Zoology, University of Cambridge, Cambridge, United Kingdom; 3 Department of Biology, University of Leicester, Leicester, United Kingdom; 4 Department of Biology, Universiteit Antwerpen, Antwerpen, Belgium; 5 Faculty of Medicine and Health Sciences, University of Nottingham, Nottingham, United Kingdom; Ghent University, Belgium

## Abstract

Desert locusts (*Schistocerca gregaria*) show an extreme form of phenotypic plasticity and can transform between a cryptic solitarious phase and a swarming gregarious phase. The two phases differ extensively in behavior, morphology and physiology but very little is known about the molecular basis of these differences. We used our recently generated Expressed Sequence Tag (EST) database derived from *S. gregaria* central nervous system (CNS) to design oligonucleotide microarrays and compare the expression of thousands of genes in the CNS of long-term gregarious and solitarious adult desert locusts. This identified 214 differentially expressed genes, of which 40% have been annotated to date. These include genes encoding proteins that are associated with CNS development and modeling, sensory perception, stress response and resistance, and fundamental cellular processes. Our microarray analysis has identified genes whose altered expression may enable locusts of either phase to deal with the different challenges they face. Genes for heat shock proteins and proteins which confer protection from infection were upregulated in gregarious locusts, which may allow them to respond to acute physiological challenges. By contrast the longer-lived solitarious locusts appear to be more strongly protected from the slowly accumulating effects of ageing by an upregulation of genes related to anti-oxidant systems, detoxification and anabolic renewal. Gregarious locusts also had a greater abundance of transcripts for proteins involved in sensory processing and in nervous system development and plasticity. Gregarious locusts live in a more complex sensory environment than solitarious locusts and may require a greater turnover of proteins involved in sensory transduction, and possibly greater neuronal plasticity.

## Introduction

**Figure 1:**
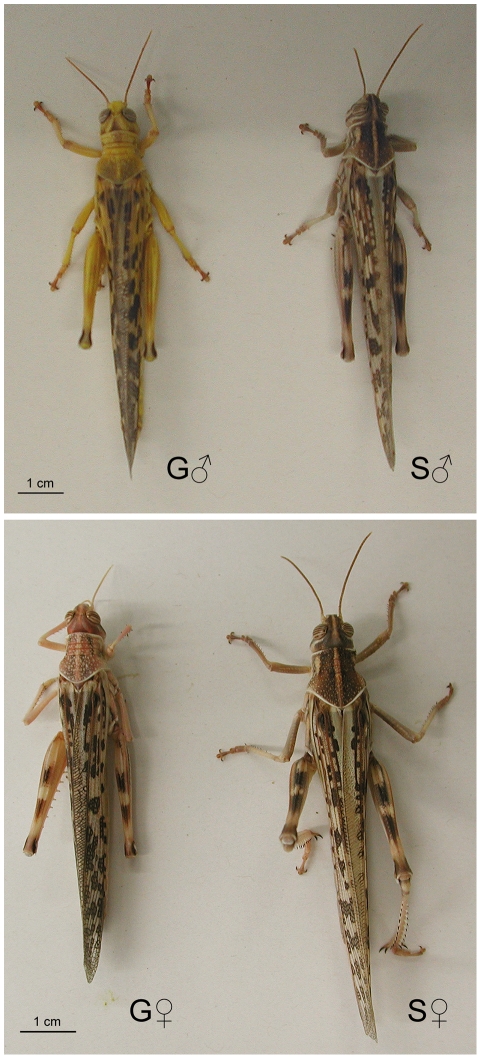
Adult gregarious and solitarious desert locusts. The upper and lower panels show males (♂) and females (♀) of the gregarious (G) or solitarious (S) phases.

Desert locusts (*Schistocerca gregaria*) are notable for their extreme form of phenotypic plasticity. Depending upon changes in population density, their genome can generate two markedly different phenotypes, which were thought to be different species when first discovered [1]; [2]. These two extreme phenotypes, called the solitarious and gregarious phases, adapt the locusts to very different lifestyles. The two phases differ in behavior, color, morphology, life history strategies, and physiology (Figure 1) [1]–[3]. Desert locusts can spend many generations in a cryptic solitarious phase in which they tend to actively avoid other locusts. Increased population density triggers the transformation towards the gregarious phase. Gregarious locusts aggregate in large migrating groups, which can ultimately lead to the formation of devastating swarms [1]–[4]. This process is fully reversible, but the full phenotype of each phase develops over the course of several generations, mediated by epigenetic mechanisms. Behavioral characteristics are amongst the first to change during phase transition. This includes the key characteristic of whether locusts are attracted or repelled by each other. In situations where solitarious locusts are unable or unwilling to avoid each other (because of restricted food supply for example), visual, olfactory (pheromonal) and mechanosensory stimuli from other locusts induce behavioral gregarization within just a few hours [5]–[7]. This behavioural change leads to a positive feedback cycle in which gregarising locusts are now constantly bombarded with stimuli from each other, which consolidates and drives further phenotypic changes.

Long-term gregarious locusts differ from long-term solitarious locusts in that they are more active, walking with the body held high off the ground, more diurnal and have a wider dietary range. By contrast, solitarious locusts are much more cryptic in behavior, walk infrequently with a characteristic low creeping gait and are more crepuscular. Long-term gregarious locusts have bright aposematic warning colors as nymphs [8] whereas solitarious locusts' cryptic behavior is complemented by cryptic green and/or brown coloration. Fully gregarious locusts have shorter hind legs, and more mechanosensory and gustatory exteroceptors on the legs; by contrast, fully solitarious locusts have more olfactory receptors on the antennae and larger eyes. Brain neuropiles involved in higher processing and learning/memory are larger in gregarious locusts, but primary sensory neuropiles are relatively larger in the brains of solitarious locusts [9]. Brain differences between phases can be detected down to the level of individual neurons involved in different sensory-motor pathways [10]–[13]. The two phases also differ in the quantities of some neuropeptides including neuroparsins and the adipokinetic hormones, although amounts can fluctuate markedly as the insects mature, and the entire phase change process is accompanied by large changes in the amounts of neurotransmitters and neuromodulators [14].

Very little is currently known about the molecular and mechanistic basis of phase polyphenism of the desert locust. Because of the primacy of behavior and therefore the CNS in desert locust phase transition [9]; [15]; [16] we recently generated a CNS Expressed Sequence Tags (EST) database which provides an extensive coverage of the *Schistocerca* CNS transcriptome [17]. This has already allowed us to identify several genes that are differentially expressed in a phase-dependent manner [17]. EST sequence information is also available for the Migratory Locust (*Locusta migratoria*) [18]; [19], a distantly related species of grasshopper that likewise shows pronounced phase polyphenism [1]; [2]. We have now developed microarrays based around these EST databases. Our locust microarray platform contained probes representing all available *S. gregaria* EST sequences contained in our database [17] and all available *S. gregaria* transcript sequences in GenBank. We also included in the microarray design all available *L. migratoria* EST sequences that have no apparent ortholog in the *S. gregaria* set. In this study we have used these microarrays to carry out a transcriptome-wide analysis of molecular differences between the CNS of adult solitarious and gregarious desert locusts, comparing the expression of thousands of genes.

## Results and Discussion

**Figure 2:**
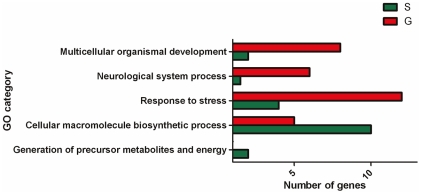
Classification of the annotated differentially expressed genes under GO categories. The number of transcripts classified under different GO terms that were more abundant in gregarious (red) and solitarious (green) locusts.

In total, 214 genes out of a total of 20,755 represented on the microarrays were differentially expressed between solitarious and gregarious locusts (at a 10% false discovery rate), 100 of which were more strongly expressed in the gregarious phase and 114 in the solitarious phase. Of the differentially expressed transcripts, 166 (78%) were identified by probe spots representing *Schistocerca* cDNAs and the remaining 48 (22%) by probes from *Locusta.* Some 40% of the differentially expressed genes have been annotated to date (Table S1), allowing them to be classified under standard ‘Gene Ontology’ (GO) categories [20] (Figure 2). By combining a Fisher's exact test (in which the set of differentially expressed genes was the test set and the complete set of annotated sequences was the reference set) and analysis of the GO term distribution, the differentially expressed genes could be classified under the five informative GO terms *Multicellular organismal development* (GO:0007275), *Neurological system process* (GO:0050877), *Response to stress* (GO:0006950), *Generation of precursor metabolites and energy* (GO:0006091) and *Cellular macromolecule biosynthetic process* (GO:0034645). In addition, differential transcript levels were also observed for genes encoding pacifastins, hexamerins, three cytochrome P450 proteins and DNA methyltransferase 2.

### *Multicellular organismal development*: nervous system development and modeling

Several of the differentially expressed genes classified under *Multicellular organismal development* are likely to be involved in nervous system development and/or remodeling. Our previous findings [17] suggested that cellular processes controlling the development and regulation of neuronal structure are more active in gregarious than in solitarious locusts. There was a higher abundance in gregarious locusts of transcripts encoding the axon path finding regulators Slit and Fasciclin. The microarray experiment confirmed the upregulation of Slit-encoding transcripts in gregarious locusts, to which can be added other phase-specific differences in genes implicated in nervous system development. These include Cullin-3 and Glia Maturation Factor (GMF), two genes implicated in actin cytoskeleton dynamics. A homolog of Cullin-3, a ubiquitin ligase scaffold protein, was more strongly expressed in gregarious locusts whereas GMF was more abundant in solitarious locusts. In *Drosophila melanogaster,* Cullin-3 indirectly controls dendritic actin rearrangements via degradation of its target, the actin-cross-linking Kelch protein [21]. Although GMFs have not yet been functionally characterized in insects, their sequences show similarity to GMFs in nematodes and mammals, where they act as factors regulating neuronal and glial growth, differentiation and regeneration [22]–[24]. They may be evolutionary conserved suppressors of the actin-related protein (Arp) 2/3 complex that enhances actin polymerization and branching [25]. The phase differences in the expression of these two genes suggest that the actin cytoskeletons of neurons in gregarious locusts are more dynamic than those of solitarious locusts.

Chromatin-remodeling is an important epigenetic mechanism that influences development through control of transcription [26]. Two genes implicated in chromatin remodeling were upregulated in gregarious locusts: Osa, a modulator of the brahma chromatin remodeling complex [27], and chromodomain helicase DNA binding protein, which appears to be a homolog of the *D. melanogaster* Mi-2 chromatin remodeler. Both are known transcriptional regulators of sensory nerve cell differentiation in *D. melanogaster* [28]; [29]. Control of mRNA splicing and translation provide a further level of developmental regulation in addition to the regulation of transcription. Homologs of the neural RNA binding proteins Musashi and Pasilla had more abundant transcript levels in gregarious locusts. Musashi is an indirect enhancer of neuronal differentiation [30]. Pasilla is a *D. melanogaster* homolog of the human Nova-1 and Nova-2 proteins [31], which are required for normal function and development of the nervous system [32], but its role in the insect nervous system is unknown.

The pronounced phase differences in the expression of genes normally implicated in nervous system development (guidance cues, regulators of cytoskeletal dynamics and transcriptional/translational controllers) are unexpected in adult animals. Although these genes have not yet been functionally characterized in locusts, our data suggest that the nervous system of gregarious locusts may show more ongoing structural remodeling than that of solitarious locusts. The key mechanisms of neuroplasticity include the alteration of existing connections and the establishment of new ones. Enhanced plasticity in the nervous system may allow gregarious locusts to adapt more easily or rapidly to new environments and deal with the sensory complexity of a swarm. This is consistent with the substantially larger brains of gregarious locusts compared to solitarious locusts. Although the primary sensory neuropils in the brain are relatively smaller in gregarious locusts, the higher integration centers are up to 50% larger than in solitarious animals [9].

A transcript that was more abundant in the solitarious CNS encodes a homolog of the Ca^2+^-activated K^+^ channel Slowpoke. In *D. melanogaster,* Slowpoke is involved in habituation [33]; [34], control of synaptic growth [35] and neurotransmitter release [36]; [37]. Reduced habituation, which may result from reduced Slowpoke expression, could be advantageous in the face of the sensory bombardment locusts receive in a swarm, as has been shown for the descending contralateral movement detector visual interneuron [11]; [13].

### Neurological system process: sensory perception

Transcripts encoding the long wavelength-sensitive opsin, a typical rhodopsin visual pigment protein [38], were more abundant in gregarious locusts. In addition, gregarious locusts showed a higher abundance of transcripts encoding a transient receptor potential-like (TRPL) channel and two different arrestins. All three kinds of proteins are key elements of the visual transduction cascade in insects [39]. Their higher expression in gregarious locusts may support vision at higher temporal resolutions. This requires a fast activation-inactivation cycle of rhodopsin, in which the inactivation of metarhodopsin is mediated through TRPL and arrestin [40]; [41].

Transcripts encoding the *S. gregaria* chemosensory proteins (CSPs) CSP1 and CSP3 [42] were more abundant in gregarious desert locusts. This observation concurs with recent findings in the migratory locust, *L. migratoria*. RNA interference against CSP3 in the gregarious phase of *L. migratoria* reduced aggregation behavior and preference for pheromones [43], with the authors suggesting that CSPs may mediate behavioral changes in response to chemosensory signals from conspecifics. Our RNA samples, however, were generated solely from the CNS, which in *L. migratoria* does not contain appreciable amounts of CSP3 transcripts [43]. Their presence and differential expression in the CNS of *S. gregaria* may point to a role for CSPs beyond that of peripheral olfaction in this species.

### Response to stress

Our data suggest that gregarious and solitarious locusts differ in some aspects of acute stress tolerance. Gregarious locusts had higher transcript levels for a thaumatin-like protein. Thaumatins were originally described as plant stress proteins where they have glucanase activity and function in a defense mechanism against pathogens [44]; [45]. They were later found in the desert locust [46] and identified in the *Tribolium castaneum* genome [47]. Since the locust and plant thaumatin proteins have a similar three-dimensional conformation, they may have similar defensive properties [46]. The greater abundance of defense proteins such as thaumatins in gregarious locusts may contribute to their increased resistance to pathogens [48], which helps to control the risk of infections spreading through the high densities of locusts found in swarms.

Gregarious animals also had higher levels of transcripts encoding heat-shock proteins (HSPs). An elevation of HSP-encoding transcripts has previously been shown in gregarious *L. migratoria* [49]. HSPs are molecular chaperones that are typically induced in response to various types of stress. They recognize proteins in a non-native conformation and have a role in the correct folding, assembly, intracellular localization, secretion, regulation and degradation of proteins [50]. Living under high population densities may lead to molecular stresses that require the increased protection provided by HSPs.

### Oxidative stress resistance

Solitarious locusts in contrast to gregarious locusts seem to invest more in oxidative stress resistance; that is, resistance to the chronic accumulation of deleterious effects that occur over a lifetime. Organisms are protected from reactive oxygen species (ROS) by antioxidant systems. Ageing is associated with an accumulation of cellular waste and the detrimental effects of ROS and other free radicals [51]. Neurons are metabolically very active and produce many ROS. Once differentiated, neurons cease dividing, leading to a rapid accumulation of ROS damage. Increased transcript levels were found in solitarious locusts for genes encoding homologs of proteins that protect against oxidative stress damage including peroxiredoxin, 5-oxoprolinase and microsomal glutathione-S-transferase. The latter is also associated with insecticide resistance [52]; [53]. Another gene displaying higher expression levels in solitarious locusts encodes a homolog of transaldolase, an enzyme in the pentose phosphate pathway, which amongst other roles has been suggested to protect neurons from free radical damage [54]. The lower expression levels of oxidative stress management genes in gregarious locusts may result in them being less resistant to oxidative stress. This, in turn, may be linked to their shorter lifespans compared to solitarious animals [1]; [2]. Lower investment in anti-oxidative mechanisms by gregarious locusts may free metabolic resources for shorter term gains, such as earlier reproduction [55].

### Basic cellular processes: protein synthesis and cellular respiration

Genes encoding homologs of proteins involved in basic cellular processes, such as protein synthesis and cellular respiration, showed higher expression in solitarious locusts. These included genes encoding electron-transferring flavoprotein dehydrogenase, mitochondrial ATP synthase coupling factor 6 (GO: *Generation of precursor metabolites and energy*), ribophorin and eight ribosomal proteins (GO: *Cellular macromolecule biosynthetic process*). This suggests that solitarious locusts, with their long lives and their enhanced protection from oxidative stress also invest more strongly in ongoing anabolic renewal than do gregarious locusts. By contrast gregarious locusts with their enhanced HSP production may instead preferentially protect already existing proteins.

### Other differentially expressed genes

Transcripts for three putative cytochrome P450 enzymes were more abundant in solitarious locusts. These enzymes are associated with detoxification and with the metabolism of a variety of compounds, including lipophilic hormones [56]. Extra sequence information and physiological assays are necessary to determine the exact identity and function of the cytochrome P450 proteins represented by the ESTs. One of them is highly similar to an enzyme that has been suggested to have ecdysone 20-hydroxylase activity in *L. migratoria* [57]; [58].

In *L. migratoria*, members of the protein superfamily containing juvenile hormone-binding proteins, hexamerins, prophenoloxidases and hemocyanins (the JHPH superfamily) are differentially expressed in the two phases [18]; [59]. We found three transcripts belonging to this family that were more abundant in the CNS of solitarious *S. gregaria*. These encoded gene products are arylphorin-like and closely resemble *L. migratoria* JHPH proteins. Arylphorins are members of the hexamerin family of proteins, which were thought simply to provide haemolymph stores of amino acids [60], but recent work suggests they have additional functions. For example, they modulate caste differentiation in termites by suppressing the transition from workers to soldiers in a juvenile hormone-responsive manner [61]; [62]. Therefore, locust JHPH proteins could have a role in the maintenance of phase-specific characteristics.

Solitarious desert locusts had higher levels of DNA methyltransferase 2 (Dnmt2)-encoding transcripts. This agrees with our previous observation of reduced Dnmt2 transcript levels following crowding of solitarious hoppers [63] and suggests that the epigenetic mechanisms that establish and/or maintain phase include alteration of the methylation state of genomic DNA [64]. Differences in the behavior of queen and worker bees have been related to different patterns of methylation in their brain DNA [65]. The Dnmt2 family can also methylate tRNAs, however [66], in which role they have been associated with stress resistance [67] and organ differentiation [68]. The differential expression of Dnmt2 could therefore reflect both phase-related differences in DNA methylation and tRNA methylation-mediated differences in stress resistance and/or CNS development.

Two pacifastin-like peptide precursors were differentially expressed. Pacifastins are a family of serine peptidase inhibitors found in arthropods [69]. The previous discovery of greater abundance of SGPP3 and SGPP4 transcripts in gregarious desert locusts using RT-qPCR [70]; [71] validates the results obtained with our microarrays.

### Non-annotated differentially expressed genes

**Figure 3:**
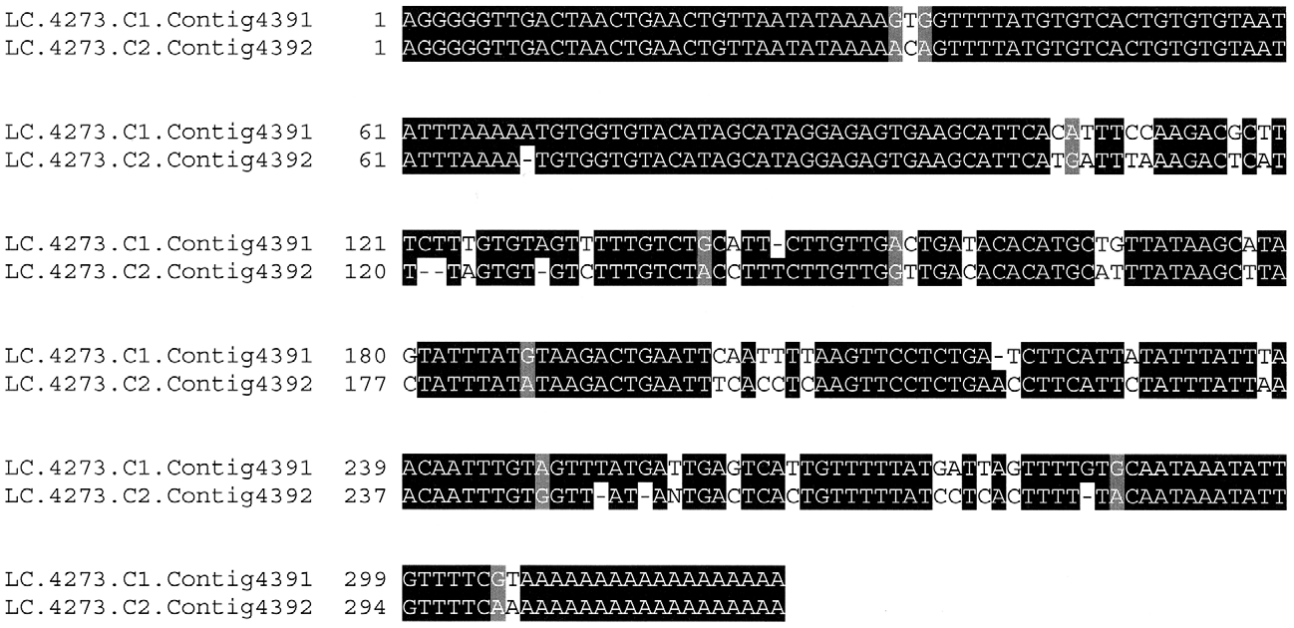
Pairwise alignment of the sequences corresponding to LC.4273.C1.Contig4391 and LC.4273.C2.Contig4392. The alignment was performed in ClustalW2 software [92]; [93] (input sequences were defined as DNA, other parameters were set at default values). Conserved residues are highlighted in black. Purine transition mutations are shaded.

Of the 214 genes that were differentially expressed in the microarray study, 128 have not yet been annotated (Table S2). Using RT-qPCR we previously identified a non-annotated gene (EST ID: LC.4273.C1.Contig4391) as having much higher expression levels in gregarious than in solitarious locusts [17]. In our present microarray experiment, this gene has the most pronounced difference in transcript abundance between the two phases (log_2_(FC) = 5.11; FC: fold change). Also present in the *S. gregaria* EST database is a highly similar sequence (86% similarity; EST ID: LC.4273.C2.Contig4392) (Figure 3). The microarray data revealed that this second transcript also occurred at higher levels in gregarious locust CNS (log_2_(FC) = 3.18). We could not determine the biological role of these transcripts based on sequence comparisons. It is probable that these transcripts do not code for proteins. Only very short ORFs (ranging in length from 3 to 7 codons) could be detected in the different reading frames and it is unclear whether these are actually protein coding or merely contain motifs consistent with a protein coding transcript. A large part of eukaryotic genomes is transcribed into non-coding RNA species (ncRNA), whilst the protein-encoding genes only make up a small portion of the genome. The biological role of only a few classes of ncRNA molecules have been determined, but many more remain to be identified and/or functionally characterized [72]. Rapid Amplification of cDNA Ends (RAcE) analyses revealed that the LC.4273.C1.Contig4391 and LC.4273.C2.Contig4392 sequences in the EST database probably represent complete transcripts. Blast searches in the non-coding RNA database [73], the functional RNA database (fRNAdb [74]) and the database of non-coding RNA families (Rfam, [75]) yielded either no or low scoring hits, but some similarities were found with sequences in the microRNA database (miRBase [76]–[80]). Whether these low scoring blast hits in non-coding RNA databases genuinely indicate homology or whether these ESTs represent new and possibly locust-specific transcripts requires further investigation. Given their markedly increased levels in the gregarious CNS, these transcripts may be part of a mechanism that establishes and/or maintains certain gregarious phase characteristics.

### General conclusions

The current microarray study revealed 214 genes that are differentially expressed between solitarious and gregarious desert locusts. Independent support for our data is provided by several previously published studies in both *S. gregaria* and *L. migratoria*. The pattern of differential gene expression in *S. gregaria* shows several similarities with that of *L. migratoria*. For example expression of transcripts for CSP3 and HSPs is stronger in gregarious locusts of both species. By contrast transcripts for hexamerins (high in gregarious *L. migratoria* heads and solitarious hind legs), cytochrome P450 proteins and alcohol dehydrogenases showed opposite patterns of expression in the two species. *S. gregaria* and *L. migratoria* belong to different sub-families of the Acrididae and have presumably evolved density dependent polyphenism independently. It remains to be seen just how strongly the convergent phase change processes are underpinned by similar changes in gene expression.

The radically different lifestyles of solitarious and gregarious locusts mean that they face very different challenges. For example, solitarious locusts rely on crypsis to avoid predation, whereas gregarious locusts in a swarm have a greatly reduced individual predation risk. Life in a swarm has its own risks however: there is increased competition for food and a higher risk of disease, and there is a need to move continually to find new resources. The challenge of extracting meaningful sensory information from the constantly moving throng of other locusts in a swarm is also considerable. The risks inherent to the solitarious lifestyle arise from their very low population densities, where for example, finding a mate or suitable oviposition sites may be difficult and require prolonged searching. Our microarray analysis has identified genes whose altered expression may enable locusts of either phase to deal with the different challenges they face. For example, genes for proteins such as HSPs that confer protection from acute physiological challenges are upregulated in gregarious locusts, as are genes encoding proteins that confer protection from infection. By contrast solitarious locusts, which require a long adult life, are more strongly protected from the slowly accumulating effects of ageing by an upregulation of genes related to anti-oxidant systems, detoxification and anabolic renewal. Gregarious locusts had a greater abundance of transcripts for proteins involved in sensory processing. Solitarious locusts have larger eyes and antennae than gregarious locusts, but the latter experience a more complex sensory environment that may require a greater turnover of proteins involved in sensory transduction, and possibly greater neuronal plasticity.

## Materials and Methods

### Locusts

The *S. gregaria* colony in Leuven originated from the Aquazoo in Düsseldorf (Germany, 1985), locusts originally from Nigeria. Gregarious locusts were reared under crowed conditions in acrylic plastic cages (40×43×85 cm) under a 13 hour light/11 hour dark photoperiod at 32°C. They were fed daily with fresh cabbage and oat flakes *ad libitum*. Solitarious locusts were reared individually for 30 successive generations in plastic boxes (15×8×7 cm), and received the same food as the crowd-reared animals. The solitarious breeding room had identical temperature and light conditions as the crowd-rearing room. For more detail on the solitarious culture, see Hoste *et al.* (2002) [81] and Berthier *et al.* (2010) [82].

### Preparation of the RNA samples

Sampling was done separately for males and females. Each condition (solitarious males or females, gregarious males or females) was represented by three independent biological samples, which each contained pooled tissues from five adult locusts aged between 2 and 10 days after final eclosion. Head ganglia (midbrain, optic lobes and subesophageal ganglion) and thoracic ganglia were dissected, cleaned and rinsed in Ringer solution (1 L: 8.766 g NaCl; 0.188 g CaCl_2_; 0.746 g KCl; 0.407 g MgCl_2_; 0.336 g NaHCO_3_; 30.807 g sucrose; 1.892 g trehalose; pH 7.2). The samples were collected in 2 ml tubes with 1.4 mm MagNa Lyser Green Beads (Roche), immediately frozen in liquid nitrogen and stored at −80°C. The samples were homogenized using a MagNA Lyser instrument (Roche) (30 s, 6500 rpm) and total RNA was extracted from the homogenates (RNeasy Lipid Tissue Mini Kit; Qiagen). A DNase treatment (RNase-free DNase set; Qiagen) was also performed to eliminate any genomic DNA contamination. The quantity and integrity of the RNA was measured spectrophotometrically on an Agilent 2100 Bioanalyzer (Agilent Technologies).

### Design of the microarrays

The locust microarrays were designed by means of the eArray system (Agilent Technologies). They contained probes representing all available *S. gregaria* transcript sequences (*i.e*. all EST sequences [17] and all *S. gregaria* transcript sequences available in GenBank), plus all *L. migratoria* EST sequences [19] having no ortholog in the *S. gregaria* set (blastn hits producing E<1E-10 were considered orthologs). Probes were designed according to the following eArray criteria: (i) probe length: 60 nucleotides, (ii) two probes per target sequence (best probe methodology) and (iii) probe orientation: sense. Other parameters were set at default settings. In total 20,755 unique locust transcript sequences (12,783 *S. gregaria* and 7,972 *L. migratoria* sequences) were represented on the arrays. Each sequence was represented by two probes, allowing for the design of the arrays in 4x44K slide format (Agilent Technologies), which also includes control features. The platform information has been deposited in the NCBI Gene Expression Omnibus (GEO) database, http://www.ncbi.nlm.nih.gov/geo) and is accessible under the accession number GPL11172.

The current microarray study aimed at identifying genes differentially expressed in the solitarious and gregarious desert locust CNS. In order to rule out sex-dependent biases in the identification of phase-specific transcriptional differences, our sampling and hybridization design were strictly balanced across the two sexes. To that purpose, the experiment consisted of two independent hybridization runs, one per sex. Each one of them involved six samples (three solitarious and three gregarious), and an n+2 A-optimal design was chosen (n = 6 samples per run), *i.e*. eight hybridizations per run were performed in a balanced and even design (see Knapen *et al.* (2009) [83].) The objective of this experimental set-up was exclusively focused on determining phase-specific differences and it was not appropriate to subsequently search for sex-specific differences.

### Labeling of the samples

Samples were labeled using the Quick Amp Labeling Kit (Two Color, Agilent), which generates fluorescent cRNA starting from total RNA or poly(dA) RNA. Each sample was labeled with Cy5 (red) and with Cy3 (green). For each sample, 2 µg total RNA was reverse transcribed by using a poly(dT) primer coupled to an antisense T7 promoter. The reverse transcriptase also catalyzes synthesis of the second cDNA strand (which contains the sense T7 promoter sequence), providing the template for synthesis and linear amplification of cRNA strands. During the transcription step Cy3- or Cy5-labeled dCTPs were incorporated in newly synthesized cRNA strands. Prior to cDNA synthesis, Spike A Mix (Agilent) was added to each sample to be labeled with Cy3 and Spike B Mix to each sample to be labeled with Cy5. These Spike-In mixes test for linearity, sensitivity and accuracy of the microarray workflow. The labeled samples were purified (RNeasy Mini Kit; Qiagen). The cRNA yield and relative amount of incorporated labeled dCTPs were determined on a NanoDrop ND-1000 UV-VIS Spectrophotometer (Thermo Scientific).

### Hybridization

Hybridizations were performed using the Gene Expression Hybridization Kit (Agilent) following the manufacturer's instructions for 17 hours at 65°C. Next, the microarrays were washed twice in GE Wash Buffer 1 (Agilent) for 1 minute each and finally in GE Wash Buffer 2 at 37°C for 1 minute.

### Scanning of the microarrays and data analysis

Microarrays were scanned using a Genepix Personal 4100A confocal scanner (Axon Instruments) at a resolution of 5 µm and excitation wavelengths of 635 nm and 532 nm. The PMT voltages for each wavelength were adjusted to obtain an overall green/red ratio as close as possible to 1. Spot identification and quantification of the fluorescent signal was carried out using GenePix Pro 6.0 software (Axon Instruments). The hybridization designs for both sexes were considered identical repeats and only phase was treated as a factor. The data obtained from all 16 hybridizations were pooled and used in a single statistical analysis (R package *limma* [84]). Spots where the median foreground intensity was less than the average local background intensity plus twice its standard deviation on all arrays were deleted before analysis [85]. Median intensities were background corrected using a normal-exponential convolution model and normalized by a Locally Weighed Scatterplot Smoothing (Loess) function [86]. For each target transcript a linear model was fitted to the intensity ratios combining the data from the two probes. The within-array replicate correlation coefficient [87] was greater than 0.84. Next, genes were ranked in order of evidence of differential expression using an empirical Bayes method [88] and solitarious-gregarious contrasts were evaluated by the linear models. False discovery rate (FDR) control was obtained by calculating adjusted p-values according to the Benjamini-Hochberg procedure [89] and a cut-off was set at a FDR of p<0.10. Annotation analysis of differentially regulated genes was performed using Blast2GO software [90]; [91], in which an annotation project for locust sequences had previously been created. All microarray data are MIAME compliant. Both raw and processed data have been deposited in the MIAME compliant NCBI Gene Expression Omnibus (GEO) database, http://www.ncbi.nlm.nih.gov/geo) and are accessible under the GEO series accession number GSE31237.

## Supporting Information

Table S1 **Annotated differentially expressed genes.** ID refers to the EST or GenBank ID; log_2_FC is the log2-transformed fold-change in expression in gregarious over solitarious CNS; *p*-values are false discovery rate (FDR)-adjusted, with a cut-off at FDR  = 10%.
(DOC)

Table S2 **Non-annotated differentially expressed genes.** ID refers to the EST or GenBank ID; log_2_FC is the log2-transformed fold-change in expression in gregarious over solitarious CNS; *p*-values are false discovery rate (FDR)-adjusted, with a cut-off at FDR  = 10%.
(DOC)
