# Supplementary material for: Microarray-Based Transcriptomic Analysis of Differences between Long-Term Gregarious and Solitarious Desert Locusts
Source: PLoS One. 2011 Nov 23;6(11):e28110. doi: 10.1371/journal.pone.0028110 (PMC3223224; doi:10.1371/journal.pone.0028110)
Supplement: Table S1 — Annotated differentially expressed genes. ID refers to the EST or GenBank ID; log2FC is the log2-transformed fold-change in expression in gregarious over solitarious CNS; p-values are false discovery rate (FDR)-adjusted, with a cut-off at FDR = 10%. (DOC) [file pone.0028110.s001.doc]

**Table S1. Annotated differentially expressed genes.**

| **ID** | **Annotation** | **Log_2_FC** | **adjusted *p*** |
| --- | --- | --- | --- |
|  |  |  |  |
| **Multicellular organismal development** | | | |
|  |  |  |  |
| LC01066A2B07.f1 | slit | 2.135 | 1.90E-03 |
| LC.696.C1.Contig810 | ankyrin repeat protein | 0.831 | 4.13E-02 |
| LMS_003043 | moleskin | 0.684 | 9.12E-04 |
| LMC_003894 | pasilla | 0.638 | 4.73E-02 |
| LC01025A2E02.f1 | osa | 0.625 | 9.35E-02 |
| LC.4225.C1.Contig4347 | cullin 3 | 0.610 | 3.14E-02 |
| LC.276.C3.Contig351 | musashi | 0.544 | 7.52E-02 |
| LC01050A1D10.f1 | chromodomain helicase dna binding protein | 0.444 | 4.14E-02 |
| LC01032B1A09.f1 | dnmt2 | -0.818 | 2.22E-02 |
| LC.1185.C1.Contig1329 | glial maturation factor | -0.879 | 8.35E-02 |
|  |  |  |  |
| **Neurological system process** | | | |
|  |  |  |  |
| gb_AF070961.1_AF070961 | Sg chemosensory protein CSP3 | 2.501 | 1.89E-03 |
| LMS_004188 | arrestin | 1.958 | 4.36E-03 |
| gb_AF070963.1_AF070963 | Sg chemosensory protein CSP1 | 1.569 | 2.22E-02 |
| LC01041B2E01.f1 | calmodulin binding protein trpl | 1.345 | 4.44E-03 |
| X80071.1 | long-wavelength sensitive opsin | 1.261 | 4.57E-02 |
| LMC_000456 | arrestin | 0.980 | 4.61E-03 |
| LC03020A2B12.f1 | calcium-activated potassium channel s.u. alpha (slowpoke) | -1.050 | 4.17E-02 |
|  |  |  |  |
| **Response to stress** | | | |
|  |  |  |  |
| LC.1083.C1.Contig1222 | heat-shock protein 70 | 2.934 | 1.26E-04 |
| LC.4562.C1.Contig4660 | heat-shock protein 20 - l2efl | 2.810 | 7.32E-05 |
| LC.1598.C1.Contig1744 | heat-shock protein 20 - l2efl | 2.297 | 5.45E-04 |
| LMC_002955 | putative defense protein | 2.073 | 9.56E-03 |
| LMS_005010 | putative defense protein | 2.041 | 9.12E-04 |
| LC.95.C1.Contig146 | small heat-shock protein | 2.011 | 2.68E-08 |
| LC01010A2C10.f1 | heat-shock protein 20 - l2efl | 1.950 | 7.03E-03 |
| gb_AY512592.1 | schistocerca thaumatin-like protein 2 | 1.850 | 8.56E-05 |
| LC.628.C1.Contig737 | dna-j hsp40 | 0.923 | 3.14E-02 |
| LC.81.C1.Contig120 | heat-shock protein 90 | 0.663 | 9.35E-02 |
| LMC_003894 | pasilla | 0.638 | 4.73E-02 |
| LC01045A2H03.f2 | dna-j homolog | 0.520 | 9.65E-02 |
| LC.3008.C1.Contig3162 | 5-oxoprolinase | -0.379 | 8.25E-02 |
| LC01066A1C11.f1 | transaldolase | -0.700 | 7.97E-02 |
| LC.2744.C1.Contig2903 | peroxiredoxin | -0.723 | 6.40E-02 |
| LMC_000724 | glutathion-s-transferase | -0.999 | 2.36E-03 |
|  |  |  |  |
| **Generation of precursor metabolites and energy** | | | |
|  |  |  |  |
| LC01031A2G01.f1 | electrontransferring flavoprotein dehydrogenase | -0.719 | 3.80E-02 |
| LC03015B1D10.f1 | mitochondrial ATP synthase coupling factor 6 | -0.854 | 3.14E-02 |
|  |  |  |  |
| **Cellular macromolecule biosynthetic process** | | | |
|  |  |  |  |
| LC01025A2E02.f1 | osa | 0.625 | 9.35E-02 |
| LC.276.C3.Contig351 | musashi (heterogeneous nuclear ribonucleoprotein) | 0.544 | 7.52E-02 |
| LC01050A1D10.f1 | chromodomain helicase dna binding protein | 0.444 | 4.14E-02 |
| LC.837.C2.Contig967 | splicing factor u2af large subunit | 0.439 | 5.07E-02 |
| LMS_006841 | translation machinery-associated | 0.399 | 2.96E-02 |
| LMC_001211 | homolog of CG-6094 | -0.307 | 9.08E-02 |
| LMC_003484 | ribosomal protein s18 | -0.496 | 9.24E-02 |
| LMC_003901 | ribosomal protein l21 | -0.518 | 1.64E-02 |
| LMS_004641 | mitochondrial ribosomal protein l34 | -0.604 | 3.75E-02 |
| LC.3174.C1.Contig3327 | ribophorin | -0.651 | 7.88E-02 |
| LMC_003667 | ribosomal protein s26 | -0.684 | 4.90E-02 |
| LC02007A1B05.f1 | 60s ribosomal protein s23 | -0.725 | 6.91E-02 |
| LMC_003028 | ribosomal protein s29 | -0.788 | 4.38E-02 |
| LMC_004314 | ribosomal protein s12 | -0.843 | 1.84E-02 |
| LMC_003149 | ribosomal protein l29 | -1.018 | 8.66E-03 |
|  |  |  |  |
| **Pacifastins** | | | |
|  |  |  |  |
| Y09606.1 | SGP3 serine protease inhibitor | 1.733 | 2.22E-02 |
| LC03003A2G07.f1 | pacifastin-related 4a - pp4a | 1.644 | 3.80E-02 |
|  |  |  |  |
| **Hexamerins** | | | |
|  |  |  |  |
| LC01046A1E10.f1 | hexamerin 2 beta | -0.775 | 5.14E-02 |
| LC.1335.C1.Contig1481 | hexamerin 1 | -0.812 | 7.77E-02 |
| LC01033A1F10.f1 | hexamerin 1 | -1.179 | 7.03E-03 |
|  |  |  |  |
| **Cytochrome P450** | | | |
|  |  |  |  |
| LC01066A1B08.f1 | putative ecdysone 20-hydroxylase (cyp450) | -0.679 | 7.97E-02 |
| LC01036B1C06.f1 | putative cytochrome p450 | -0.849 | 2.88E-02 |
| LC03012A1C01.f1 | cytochrome p450 cyp6bk17 | -1.065 | 4.74E-02 |
|  |  |  |  |
| **Other** | | | |
|  |  |  |  |
| LC.2162.C1.Contig2323 | epsilon-trimethyllysine 2-oxoglutarate dioxygenase | 1.735 | 5.93E-03 |
| LC.1009.C1.Contig1150 | 3-oxoacyl-[acyl-carrier-protein] reductase 1 | 1.431 | 4.16E-03 |
| LC.1325.C1.Contig1470 | glucose transporter (sugar transporter) | 0.718 | 5.26E-02 |
| LC01042A1D05.f1 | calcyclin binding protein | 0.608 | 4.12E-02 |
| LC01042B1G05.f1 | nefa-interacting nuclear protein nip30 | 0.577 | 7.99E-02 |
| LC01027A1B11.f1 | heterogeneous nuclear ribonucleoprotein u-like 1 | 0.567 | 9.20E-04 |
| LC03022A2F04.f1 | pmp22 peroxisomal membrane | 0.535 | 1.01E-02 |
| LC.975.C1.Contig1112 | sugar transporter | 0.521 | 8.27E-02 |
| LMS_005336 | lipase | 0.436 | 8.96E-02 |
| LC01026A1H09.f1 | cation efflux protein zinc transporter | 0.403 | 2.55E-02 |
| LMC_003614 | x-prolyl aminopeptidase (aminopeptidase p) soluble | -0.374 | 6.00E-02 |
| LMS_006838 | thump domain containing 3 | -0.390 | 8.66E-03 |
| LC.1161.C1.Contig1303 | transmembrane gtpase | -0.433 | 7.25E-02 |
| LMS_002926 | reverse transcriptase | -0.436 | 5.14E-02 |
| LMS_007100 | aldo-keto reductase | -0.468 | 9.17E-02 |
| LC.4152.C1.Contig4279 | nascent polypeptide associated complex protein alpha subunit | -0.472 | 9.08E-02 |
| LMC_002631 | proteasome (macropain) alpha 2 | -0.485 | 7.97E-02 |
| LC.1332.C1.Contig1478 | solute carrier family 3 | -0.507 | 8.73E-02 |
| LC.3244.C1.Contig3389 | comm domain containing 4 | -0.537 | 3.02E-03 |
| LC.682.C1.Contig796 | mpv17 mitochondrial membrane protein-like | -0.584 | 8.27E-02 |
| LC03002A2E03.f1 | ribosome-associated membrane protein | -0.637 | 8.02E-03 |
| LC.28.C3.Contig37 | dead box atp-dependent rna helicase | -0.791 | 9.43E-02 |
| LC.317.C1.Contig399 | molybdopterin cofactor sulfurase | -0.806 | 3.15E-02 |
| LC01020B2F10.f1 | abc transporter | -0.830 | 3.80E-02 |
| LC.4385.C1.Contig4496 | zinc-containing alcohol dehydrogenase | -0.888 | 8.98E-02 |
| LC.2581.C1.Contig2743 | dipeptidyl-peptidase | -0.918 | 8.59E-02 |
| LC.4675.C1.Contig4764 | phospholipase b domain containing 2 | -0.974 | 1.12E-02 |
| LC01034B1D08.f1 | alcohol dehydrogenase | -1.218 | 4.45E-02 |
| LMS_006542 | serine 3-dehydrogenase | -1.325 | 4.16E-03 |
| LC.388.C1.Contig470 | alcohol dehydrogenase | -1.770 | 3.91E-02 |
