# Supplementary material for: Microarray-Based Transcriptomic Analysis of Differences between Long-Term Gregarious and Solitarious Desert Locusts
Source: PLoS One. 2011 Nov 23;6(11):e28110. doi: 10.1371/journal.pone.0028110 (PMC3223224; doi:10.1371/journal.pone.0028110)
Supplement: Table S2 — Non-annotated differentially expressed genes. ID refers to the EST or GenBank ID; log2FC is the log2-transformed fold-change in expression in gregarious over solitarious CNS; p-values are false discovery rate (FDR)-adjusted, with a cut-off at FDR = 10%. (DOC) [file pone.0028110.s002.doc]

**Table S2. Non-annotated differentially expressed genes.**

| **ID** | **Log_2_FC** | **adjusted *p*** |
| --- | --- | --- |
| LC.4273.C1.Contig4391 | 5.112 | 5.28E-06 |
| LC.164.C1.Contig229 | 3.664 | 1.40E-04 |
| LC.4273.C2.Contig4392 | 3.187 | 7.29E-05 |
| LMC_000579 | 2.790 | 5.10E-06 |
| LC03013B1A09.f1 | 2.648 | 2.50E-07 |
| LC.1930.C2.Contig2086 | 1.942 | 3.35E-06 |
| LC03008B2E11.f1 | 1.929 | 1.10E-02 |
| LC.4391.C1.Contig4502 | 1.821 | 1.28E-03 |
| LC03001A1E06.f1 | 1.617 | 2.88E-02 |
| LC.1413.C1.Contig1558 | 1.612 | 6.58E-03 |
| LC.4015.C1.Contig4142 | 1.593 | 1.01E-02 |
| LC.4671.C1.Contig4760 | 1.549 | 1.82E-04 |
| LMS_003750 | 1.517 | 7.87E-02 |
| LC03016B2G08.f1 | 1.334 | 3.14E-02 |
| LC.4653.C1.Contig4743 | 1.311 | 1.84E-02 |
| LC03020B2G08.f1 | 1.256 | 2.36E-03 |
| LC01053A1F07.f1 | 1.186 | 3.72E-05 |
| LC01053A1F05.f1 | 1.156 | 1.07E-03 |
| LC.2165.C1.Contig2326 | 1.135 | 2.37E-02 |
| LC01058A2H04.f2 | 1.126 | 8.83E-02 |
| LC.4521.C1.Contig4621 | 1.076 | 4.61E-03 |
| LC.1930.C1.Contig2085 | 1.020 | 3.85E-02 |
| LC02007A2G10.f1 | 0.970 | 1.33E-02 |
| LC01058A1C06.f1 | 0.966 | 3.68E-03 |
| LMC_001631 | 0.958 | 1.23E-03 |
| LC02007B2B05.f1 | 0.890 | 1.68E-02 |
| LC.1147.C1.Contig1289 | 0.885 | 9.95E-03 |
| LC.260.C2.Contig333 | 0.823 | 6.00E-02 |
| LC.115.C1.Contig169 | 0.790 | 4.50E-04 |
| LC01017B2A01.f1 | 0.786 | 7.43E-02 |
| LMC_002459 | 0.778 | 5.60E-06 |
| LC01057A2G01.f1 | 0.775 | 9.80E-02 |
| LC.2927.C1.Contig3087 | 0.723 | 2.74E-04 |
| LC.2544.C1.Contig2706 | 0.719 | 4.17E-02 |
| LMS_004398 | 0.717 | 1.90E-03 |
| LC03019B2G09.f1 | 0.714 | 9.08E-02 |
| LC.1861.C1.Contig2019 | 0.708 | 4.39E-03 |
| LC01063B1C04.f2 | 0.695 | 4.38E-02 |
| LC03013A1G11.f1 | 0.684 | 4.36E-03 |
| LC01028A1G04.f1 | 0.674 | 9.17E-02 |
| LC03005A2H01.f1 | 0.633 | 1.84E-03 |
| LC03009A2G02.f1 | 0.631 | 8.29E-02 |
| LMC_001092 | 0.629 | 3.14E-02 |
| LC03013B1D12.f1 | 0.625 | 3.53E-03 |
| LC.2380.C1.Contig2547 | 0.621 | 6.48E-02 |
| LMS_000472 | 0.606 | 1.37E-02 |
| LMS_003895 | 0.597 | 1.29E-02 |
| LC01055B1G07.f2 | 0.576 | 4.62E-03 |
| LC01049A2D04.f1 | 0.541 | 4.57E-02 |
| LC.3366.C1.Contig3505 | 0.537 | 6.61E-03 |
| LMS_001249 | 0.532 | 9.69E-02 |
| LMS_006047 | 0.523 | 1.89E-03 |
| LC.4355.C1.Contig4470 | 0.521 | 9.80E-02 |
| LC02007B2E08.f1 | 0.517 | 3.75E-03 |
| LMS_003144 | 0.497 | 5.24E-02 |
| LMC_001197 | 0.464 | 4.34E-02 |
| LC03016A1B08.f1 | 0.455 | 9.08E-02 |
| LMC_003785 | 0.450 | 3.11E-02 |
| LC01019A2H02.f1 | 0.447 | 4.46E-02 |
| LC01051A2F08.f3 | 0.438 | 4.61E-03 |
| LMC_000158 | 0.370 | 6.01E-02 |
| LC.4031.C1.Contig4158 | -0.328 | 4.57E-02 |
| LC03015A1C03.f1 | -0.367 | 8.26E-02 |
| LC01023B1F02.f1 | -0.381 | 3.21E-02 |
| LC03019B1B05.f1 | -0.394 | 9.80E-02 |
| LC.3409.C1.Contig3547 | -0.409 | 4.01E-02 |
| LC03003A2C01.f1 | -0.416 | 3.14E-02 |
| LMC_004370 | -0.420 | 6.91E-02 |
| LC03006B2A01.f1 | -0.423 | 9.08E-02 |
| LMS_000338 | -0.426 | 1.25E-02 |
| LC03021A2A04.f1 | -0.429 | 6.40E-02 |
| LC.4146.C1.Contig4273 | -0.439 | 6.08E-02 |
| LC.3011.C1.Contig3165 | -0.452 | 3.80E-02 |
| LMS_003354 | -0.455 | 8.21E-03 |
| LC.3839.C1.Contig3966 | -0.457 | 3.80E-02 |
| LC.2850.C1.Contig3010 | -0.459 | 9.08E-02 |
| LMS_003575 | -0.461 | 3.48E-02 |
| LMS_005343 | -0.466 | 2.62E-02 |
| LC01013A2E09.f1 | -0.483 | 1.72E-02 |
| LMC_003364 | -0.494 | 6.29E-02 |
| LC.107.C1.Contig160 | -0.513 | 4.46E-02 |
| LC03023B1H09.f1 | -0.517 | 2.22E-02 |
| LC03009B2F04.f1 | -0.521 | 2.88E-02 |
| LMS_003122 | -0.538 | 4.74E-02 |
| LC01023A1H12.f1 | -0.548 | 9.17E-02 |
| LC03013A2B09.f1 | -0.553 | 5.50E-03 |
| LMC_001405 | -0.555 | 6.69E-03 |
| LC03019B1F12.f1 | -0.571 | 6.01E-02 |
| LMS_003072 | -0.575 | 1.43E-02 |
| LMC_003363 | -0.579 | 3.56E-03 |
| LC03022B2E02.f1 | -0.582 | 3.10E-02 |
| LC01031A1D02.f1 | -0.611 | 1.53E-02 |
| LC03014A1E06.f1 | -0.615 | 1.12E-02 |
| LC03021A1G09.f1 | -0.618 | 5.69E-02 |
| LC01005B1B06.f1 | -0.629 | 2.94E-02 |
| LMS_001742 | -0.632 | 2.41E-02 |
| LC01056A1F08.f2 | -0.636 | 4.57E-02 |
| LC03022A1C06.f1 | -0.641 | 1.68E-02 |
| LC03012B2H04.f1 | -0.643 | 3.45E-03 |
| LC03023B2G04.f1 | -0.646 | 2.36E-03 |
| LC01019A2E11.f1 | -0.651 | 8.56E-03 |
| LC03020B2G03.f1 | -0.657 | 5.21E-03 |
| LC.4394.C1.Contig4505 | -0.684 | 8.96E-02 |
| LC03008A1H08.f1 | -0.691 | 2.47E-02 |
| LC.1917.C1.Contig2073 | -0.704 | 7.62E-02 |
| LC03001A2A06.f1 | -0.712 | 9.65E-02 |
| LC.4233.C1.Contig4354 | -0.732 | 3.04E-05 |
| LC.1882.C1.Contig2039 | -0.820 | 1.23E-03 |
| LC03014A2E03.f1 | -0.904 | 9.91E-02 |
| LC03007A1E12.f1 | -0.905 | 3.65E-03 |
| LC.2144.C1.Contig2306 | -0.913 | 3.80E-02 |
| LC.4292.C1.Contig4411 | -0.951 | 7.99E-02 |
| LC03014A2G02.f1 | -0.951 | 6.66E-03 |
| LC03001A1F11.f1 | -0.996 | 4.12E-02 |
| LC01010A2A12.f1 | -1.026 | 4.61E-03 |
| LC.2283.C1.Contig2446 | -1.065 | 9.35E-02 |
| LC03010A2B10.f1 | -1.099 | 8.96E-02 |
| LC03016A1F10.f1 | -1.104 | 2.37E-02 |
| LC03003B2G06.f1 | -1.155 | 3.24E-02 |
| LC03001B2C05.f1 | -1.176 | 4.36E-03 |
| LC01035B2F03.f1 | -1.184 | 3.24E-02 |
| LC.3724.C1.Contig3855 | -1.194 | 3.80E-02 |
| LC01012B2D11.f1 | -1.201 | 1.17E-02 |
| LC.3768.C1.Contig3898 | -1.321 | 3.92E-04 |
| LC03004B1G06.f1 | -1.339 | 5.21E-03 |
| LC03008B1B03.f1 | -1.474 | 5.73E-07 |
| LC03004A1E04.f1 | -1.673 | 2.74E-04 |
| LC.3509.C1.Contig3647 | -2.116 | 7.63E-07 |
